# The Effects of a Senofilcon A Contact Lens With and Without a Photochromic Additive on Positive Dysphotopsia Across Age

**DOI:** 10.1097/ICL.0000000000000731

**Published:** 2020-07-28

**Authors:** Billy R. Hammond, John Buch, Leilani Sonoda, Lisa Renzi-Hammond

**Affiliations:** Department of Psychology (B.R.H.), Vision Sciences Laboratory, Behavioral and Brain Sciences Program, The University of Georgia, Athens, GA; Johnson and Johnson Vision Care, Inc (L.S.), Jacksonville, FL; and Department of Health Promotion and Behavior (L.R.-H.), Human Biofactors Laboratory, Institute of Gerontology, College of Public Health, The University of Georgia, Athens, GA.

**Keywords:** Glare, Scatter, Positive dysphotopsia, Contact lens, Photochromic, Two-point thresholds

## Abstract

**Objective:**

The visual effects of wearing a photochromic contact lens (test) were directly compared with a nonphotochromic contact lens (control). Positive dysphotopsia (halos, starbursts) and intraocular scatter (behaviorally determined) were assessed. Both younger and middle-aged subjects were evaluated to examine the influence of age.

**Methods:**

Fifty-four subjects (18–62 years) were tested using a contralateral design. Subjects were fit with a photochromic contact lens on one eye and a nonphotochromic contact lens on the other eye, randomly assigned. Testing occurred with and without photochromic activation (darkened) by use of a violet activator (365 nm, half-bandwidth 20 nm). The extent of dysphotopsia (halos and spokes) was measured using an aperture (∼4 mm) that created a bright point source of light 45 inches from the plane of the eye. Between the point source and subject, a centering precision caliper was used to measure lateral spread. Two-point thresholds were determined by measuring the minimum distance between two points of broadband xenon light.

**Results:**

The photochromic contact lens produced smaller halo diameters than the control contact lens, both activated (41% on average) and inactivated (21% on average), and age strata was a significant factor (*P*<0.001) with the older group showing a greater reduction. The photochromic contact lens produced smaller starburst diameters than the control contact lens, both activated (37% on average) and inactivated (23% on average), and age strata was a significant factor (*P*=0.001) with the older group showing a greater reduction. The two-point thresholds were reduced (25% activated, 9% inactivated) on average but the age effect was not significant (*P*<0.10).

**Conclusions:**

The senofilcon A lens with photochromic additive reduced the extent of positive dysphotopsia compared with the same lens without the additive, regardless whether the lens was activated or not. The visual benefit was greatest with the older subjects.

A recent innovation in the design of soft contact lenses is the introduction of a photochromic additive.^1,2^ This photochromic is a highly conjugated compound, which exists in an equilibrium mixture of closed (inactivated) and open (activated) forms. The equilibrium between the two forms is driven by the intensity and wavelength of the incident light (and the thermal conditions favoring the forward or reverse reactions via Claisen rearrangement).^3,4^ When not in the presence of an activating light source, the photochromic still absorbs ultraviolet (UV) and high energy visible (HEV) light (Fig. 1 in Renzi-Hammond et al.^1^ 2019). Once UV and/or HEV light is absorbed, the photochromic compound equilibrium shifts to the activated state which extends its filtering within the visible light range. This filtering continues until the UV and/or HEV light is minimized and the molecule relaxes to its preferred lower-energy equilibrium state (largely transparent). This balance between the inactivated and activated state of the photochromic is always in flux and reflects the ambient energy incident on the lens and, to a lesser extent, the local and ambient temperature of the contact lens being influenced by ocular temperature. The only delay in these kinetics is based on the time necessary for the photochromic molecules to fully change shape and/or relax; with normal use (say, walking outdoors on a sunny day) this would be around 45 s to reach half maximum absorbance and around 90 sec to fade back half way from the fully saturated absorbance. The goal of this design was to create a photochromic contact lens that would adjust to the ambient light milieu in a manner consistent with how the system naturally adapts to the changing light levels an individual experiences in a typical day.

**FIG. 1.**
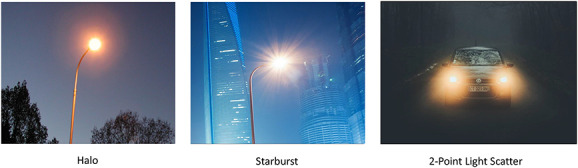
Photographic images illustrating the effects of light halos, spokes, and two-point light scatter.

Decreasing sensitivity or increasing filtering based on light intensity has some predictable effects on visual function. Photochromic compounds in plastic spectacle lenses were first introduced by Corning in 1962 (PhotoGray; Patent US3208860A), who later developed them as part of their “GlareControl” line for medical filter applications. Such lenses would later be marketed by many companies as glasses that could serve as regular prescription spectacles and sunglasses. This latter use was based on the obvious idea that filtering could reduce the negative visual effects associated with the scattering of bright light (discomfort and disability glare).^5^ Adjustable filters (e.g., the photochromic cornea of some fish),^6^ such as changing visual sensitivity, are at least one natural approach to dealing with variations in light levels in other species. Filtering intense light can have the added benefit of cleaning up the signal (e.g., reducing light scatter; effectively reducing the spread in the point spread function^7^). This is illustrated in Figures 1 and 2.

**FIG. 2.**
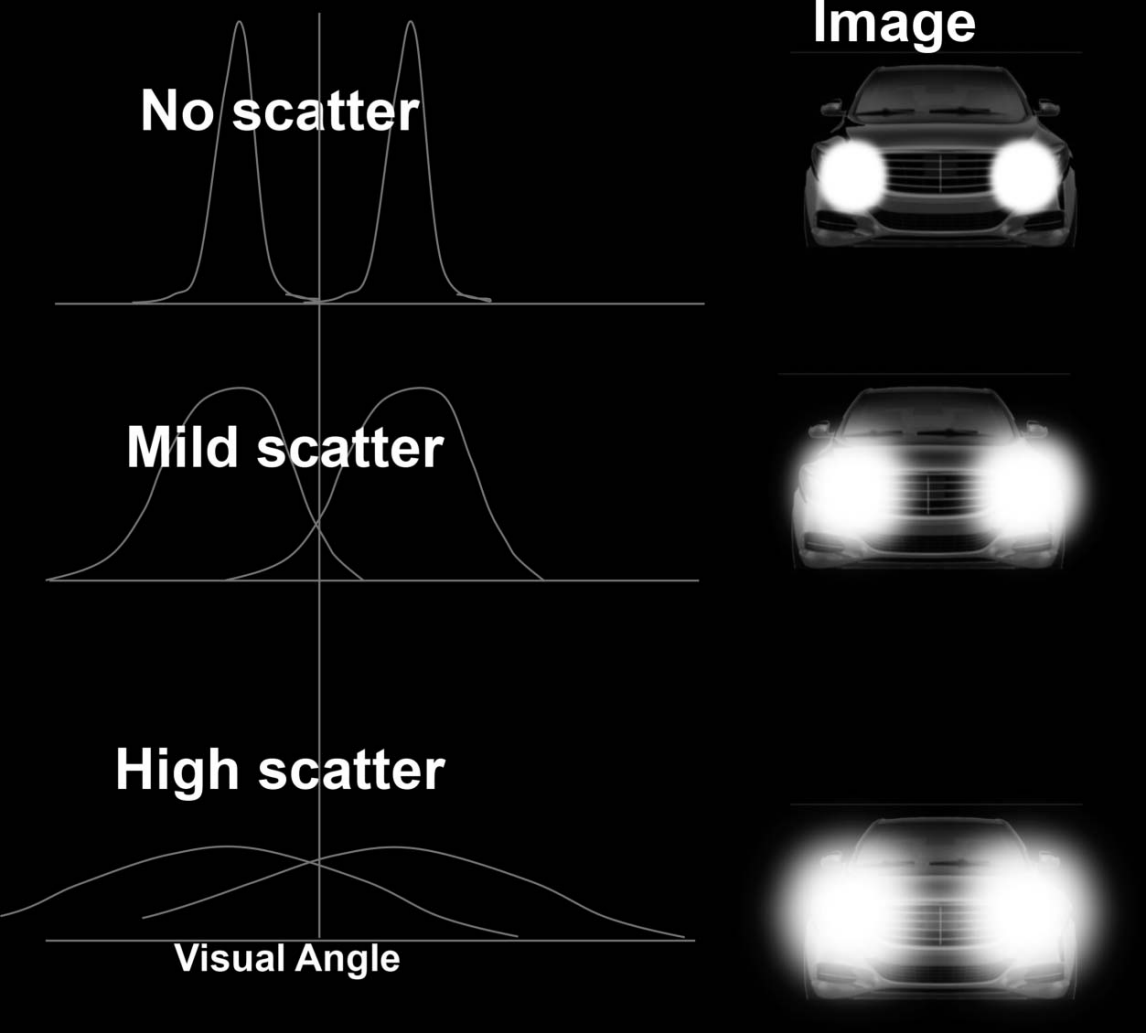
An illustration of how measuring the separation of two bright points of light (illustrated as car headlights) is a behavioral measure of the width of the point spread function.

Many effects of light scatter are subtle, especially during the day, but some are obvious, particularly with bright light and at night. For example, when viewing a point source (ranging from the bright light of the sun, street or headlights to the dim glow of a distant star) light will spread in two distinct, and independent, patterns^8^ often referred to as halos and starbursts. Halos are primarily the result of forward light scatter arising primarily from the crystalline lens. Starbursts (essentially ciliary corona) stem largely from diffraction and aberrations that arise from small particle scattering along the optical path.^9^ With broadband light, the effect is needle-like spokes radiating from the image of the source. These visual perturbations are known to occur with high frequency as a result of ocular issues such as laser corrections or cataracts (and some Intraocular lens).^10^ The association with ocular problems, however, is simply a reflection of their magnitude. To a certain degree, predicted by degree of ocular inhomogeneity's, they are present in all eyes. It follows that, with age and accumulated error (e.g., increasing opacification of the crystalline lens), the nature of these visual effects would also change. The nature of the change is not obvious. For example, older lenses scatter more, but aberrations tend to come from the edges and older subjects have smaller pupils. In this study, we assessed a young and older (mostly middle-aged) group to determine whether age and photochromic filtering interact to influence the geometry of glare.

## METHODS

### Subjects

A total of 62 subjects were enrolled from a single clinical site in this study (Georgia Center for Sight, Greensboro, GA) ranging in age from 18 to 62 years. A prospective, randomized, subject-masked, contralateral design was used. Subjects were required to be adapted wearers of senofilcon A or other spherical silicone hydrogel soft contact lenses. All subjects were required to have best-corrected visual acuity of 20/25 or better in each eye with the correction being limited to the range of −1.00 to −4.50 D. Subjects were excluded if ocular/systemic issues were reported that could interfere with testing or contact lens wear, such as corneal distortion from previous hard or rigid gas permeable contact lens wear. These issues were evaluated by the attending clinician (e.g., subjects were ineligible if there were grade three or higher using the FDA Slit Lamp Classification Scale). Iris color was based on comparison, by a single experienced observer, to a standard scale.^11^

Of those enrolled, 60 (96.8%) subjects were assigned and administered at least one study lens, whereas 2 (3.2%) subjects were screen failures and/or not assigned. Of the total assigned subjects, all 60 (96.8%) subjects completed the study and were analyzed for safety. Six subjects were excluded from cohort (e.g., protocol noncompliance (4), missing data (2)), resulting in 54 subjects that comprised the per-protocol population (Table 1) and were analyzed for efficacy.

**TABLE 1.** Per-Protocol Population Demographics

	Age Group: 18–39 years, N=35	Age Group: 40–65 years, N=19	Overall, N=54
Gender n (%)			
Female	28 (80.0)	18 (94.7)	46 (85.2)
Male	7 (20.0)	1 (5.3)	8 (14.8)
Total	35	19	54
Race n (%)			
American Indian or Alaska native	0 (0.0)	0 (0.0)	0 (0.0)
Asian	0 (0.0)	0 (0.0)	0 (0.0)
Black or African American	13 (37.1)	8 (42.1)	21 (38.9)
Native Hawaiian or other Pacific Islander	0 (0.0)	0 (0.0)	0 (0.0)
White	22 (62.9)	10 (52.6)	32 (59.3)
Other	0 (0.0)	1 (5.3)	1 (1.9)
Total	35	19	54
Ethnicity n (%)			
Hispanic or Latino	1 (2.9)	1 (5.3)	2 (3.7)
Non-Hispanic or Latino	34 (97.1)	18 (94.7)	52 (96.3)
Total	35	19	54
Iris category n (%)			
Dark iris	25 (71.4)	11 (57.9)	36 (66.7)
Light iris	10 (28.6)	8 (42.1)	18 (33.3)
Total	35	19	54
Age (y)			
n	35	19	54
Mean (SD)	28.0 (6.28)	47.6 (5.93)	34.9 (11.24)
Median	28.0	48.0	34.0
Min–Max	18.0–39.0	40.0–62.0	18.0–62.0

The study was performed in accordance with ISO 14155:2011 (Clinical investigation of medical devices for human subjects) and followed the tenets of the Declaration of Helsinki. Verbal and written informed consent were obtained from all subjects and the protocols were approved by the Sterling Institutional Review Board, Atlanta, GA.

## MATERIALS AND METHODS

### Experimental Test and Control Contact Lenses

This study evaluated senofilcon A contact lenses with (test) and without (control) a photochromic additive. These lenses were provided by Johnson & Johnson Vision Care, Inc (Jacksonville, FL). Differences in visual performance between the control lens and the test lens were measured during a single clinic visit.

Halo diameter, starburst diameter, and 2-point light separation were all performed using broad-band white light (simulated sunlight; Fig. 2 in Renzi-Hammond and Hammond, 2016)^5^ with and without a violet activator for both test and control lenses. In addition, the 2-point light separation test was performed with and without a 403±8-nm band-pass filter. This short-wavelength condition was added because of the significant effects of age on short-wave scatter arising from the lens.^12^ The activator was used to cause a steady-state activation of the photochromic test lens during testing and was used in the control condition simply to keep conditions consistent between the two eyes. Both lens type and activation state were randomized within subjects. With respect to lens type, the test and control lenses were randomized within each subject, to OD-Test/OS-Control, or OD-Control/OS-Test; activated for first measure, or inactivated for first measure. When the randomization scheme resulted in the initial test in the activated state, 10 min of washout time was given before any additional testing on the lens, to ensure that the photochromic test lens could return to its baseline state. When the violet activator was used to activate the lens, the participant was exposed to the activator for 60 sec before visual function testing, and the visual function measures were completed with the activating light on.

### Activation of the Photochromic

Activation of the photochromic was achieved using a violet activator consisting of Light-emitting diode (LEDs) (peak λ=365 nm, half bandpass=20 nm). One activator was placed on each of the temporal sides of the head and chin rest assembly, so that each eye received light from the temporal side. The activation light beam had a dispersion angle that covered approximately 2-inches square. The test eye was always monitored using a bore camera (TechFx). The violet activation LEDs were kept at a low constant rate (9.22 μW/cm^2^/nm) whereas all of the visual measures (two-point thresholds, halo and starburst sizes) were collected in both conditions (the test and control lens), so that visual function measurements were made using the same amount of light in the activated state, even if the control lens was being tested.

We estimated the degree of activation of the photochromic by measuring the spectral sensitivity in the eye wearing the activated photochromic test lens and the other eye wearing the control lens (using identical conditions). Taking the difference of the two spectral sensitivity curves yielded a difference spectrum. Based on these measures (relatively rough, because it assumes homogeneity across the two eyes), we estimated that the photochromic was maximally activated (bench measurements suggest that the maximum optical density for the lens is around 0.40).

### Apparatus

A schematic of the optical system can be found in Figure 2 of Hammond et al.^2^ A 1000W Xenon arc lamp (an approximation of broadband noonday sunlight) was used as the primary light source. Achromatic lenses relayed the light to provide homogeneous back illumination of an opaque light shield with a collapsible baffle (a center piece lowered as the apertures spread to make distinct light points that were physically disconnected). This light shield contained two small (2 mm) apertures that could be brought together into a single point or slowly moved apart as two separate points (45 inches from the plane of the eye). A digital micrometer was used to measure these lateral movements during two-point threshold testing (∼23 inches from the plane of the eye). When measuring halo and starburst sizes, the same light source arrangement was used, but the stimulus was always presented as a single point of light. Stabilization of the subject's head was accomplished by an adjustable chin and forehead rest assembly. The position of the eye was constantly monitored by a small-bore camera to ensure proper alignment with the test stimuli (the opposing eye was always patched).

### Procedure

All visual function testing was conducted on the same apparatus (described above), in a darkened room. Individual test procedures were as follows:

### Two-Point Thresholds

Light from the point source (i.e., light emerging from the 2 mm apertures in the light shield) was focused by a long (220 mm) focal length lens (17.8 cm from the plane of the eye) so that the subject's eye was in partial Maxwellian view (meaning the eye was in the focusing beam but the plane of the eye was anterior to the final focal point). When the points were maximally close together, the stimulus appeared as a single, bright point of light. The two apertures were slowly moved apart (the distance was quantified with a digital micrometer, centered on the apertures, that was part of the physical structure forming the apertures) from this “zero point,” and subjects indicated when the spread from each light point did not overlap (e.g., when they first perceived a small black space between the two points). Three trials were completed for each lens type, in each activation state, in each of two test conditions, for a total of 24 total trials for each subject. As stated previously, lens order and activation state were randomized for each subject, with appropriate washout periods. In condition 1, two-point thresholds (the minimum distance between the apertures where participants could distinguish the points as separate) were measured using only the white light from the xenon arc source (attenuated with neutral density filters to about 10 cd/m2 measured at the aperture). In condition 2, the neutral density filters were removed and replaced with a 403-nm narrow bandpass (8-nm half-bandpass) interference filter. This filter significantly reduced the energy of the stimulus (from 32 lux at the light shield to 0.3 lux), but increased the spread of the light.

### Halo and Starburst Diameters

Before testing, subjects were given a visual guide that provided examples of what halos and starbursts commonly look like to normal viewers in natural scenes (e.g., off of car headlight, streetlights, against a blue sky or created through stadium lights). Glare geometry (halo and starburst sizes) was measured using an aperture (∼4 mm at the light shield) that created a bright point source of light 110 cm from the plane of the eye. Between the point source and subject, a centering precision caliper (approximately 58 cm from the plane of the eye) was used to measure lateral spread of halos (diffusion around the source) and visual spokes of the resulting starburst. For the caliper guides to be clearly seen by the subjects, the guides were covered with highly reflective material. A dim white LED was affixed to the head rest assembly above the subject's head that illuminated the arms of the calipers.

The ascending and descending method of limits was used to determine the diameters of the halo (diffuse light around the point source) and starburst (spokes radiating outward from the point source). A single trained experimenter moved the caliper guides until subjects indicated that the guides just surrounded the halo and the starburst. Participants completed three trials for halos and three trials for starbursts, in each activation state, with each lens. Consequently, each participant completed a total of 12 trials with the test lens, and 12 trials with the control lens for starbursts, and 12 trials with the test lens, and 12 trials with the control lens for halos.

Photometric calibrations (both in the visible and UV) were performed using an ILT950 spectroradiometer (International Light Technologies, Peabody MA). Wedge and neutral density radiometric calibrations were performed using a Graseby Optronics United Detection Technology (UDT) instrument (Orlando, FL). The same UDT instrument was used before every experimental session to ensure that the total light output of the optical system remained constant and consistent throughout the study.

## RESULTS

Subjects (n=54, tested per protocol) from ages 18 to 62 years were assessed. Three endpoints were analyzed by lens and by age and are summarized in Table 2. For each variable, higher values suggest reduced performance (e.g., an increased value for the two-point thresholds indicate more spread/scatter). No covariation with iris pigmentation was seen for any of the analyses.

**TABLE 2.** Least Square Mean Difference Estimates and Adjusted Confidence Intervals by Age Group

Variable	Age	Comparison	LS Mean Estimate	SE	Adjusted Lower CL	Adjusted Upper CL	Test Lens Out-Performs Control?
2-Point light spread function (broad-band white)	18–39	Activated (test − control)	−0.97	0.19	−1.46	−0.49	Yes
		Inactivated (test − control)	−0.86	0.19	−1.34	−0.37	Yes
2-Point light spread function (403 NM)	18–39	Activated (test − control)	−1.10	0.31	−1.90	−0.30	Yes
		Inactivated (test − control)	−0.91	0.31	−1.71	−0.11	Yes
2-Point light spread function (broad-band white)	40–65	Activated (test − control)	−1.29	0.26	−1.95	−0.63	Yes
		Inactivated (test − control)	−0.51	0.26	−1.17	0.15	No
2-Point light spread function (403 NM)	40–65	Activated (test − control)	−1.82	0.43	−2.90	−0.73	Yes
		Inactivated (test − control)	−1.18	0.43	−2.27	−0.10	Yes
HALOS	18–39	Activated (test − control)	−19.65	2.58	−26.14	−13.15	Yes
		Inactivated (test − control)	−4.74	2.58	−11.24	1.75	No
HALOS	40–65	Activated (test − control)	−30.21	3.5	−39.02	−21.39	Yes
		Inactivated (test − control)	−12.97	3.5	−21.78	−4.15	Yes
Spokes	18–39	Activated (test − control)	−26.83	3.03	−34.38	−19.29	Yes
		Inactivated (test − control)	−10.14	3.03	−17.68	−2.59	Yes
Spokes	40–65	Activated (test − control)	−34.28	4.11	−44.52	−24.04	Yes
		Inactivated (test − control)	−20.62	4.11	−30.86	−10.38	Yes

Family-wise alpha was 0.05 for all comparisons Significantly lower differences were concluded if the upper limit of the 95% CI was below 0. Comparisons within age groups were adjusted for multiplicity using a simulation adjustment with family wise alpha =0.05.

CL, confidence limits; SE, standard error.

### Two-Point Light Threshold without and with 403-nm Band-Pass Filter

The minimum distance (mm) required to resolve two points of light as distinct were measured under various conditions (e.g., with and without an activating light source, and with and without a 403 nm band pass filter—thus eight testing scenarios per age group). In all cases, the photochromic lens provided a shorter distance between the two points of light than the nonphotochromic lens. All but one of these conditions (older group/inactivated/without filter) were statistically significant. Predictably, the 403-nm band-pass filter created greater scattering overall. It also created the largest separation between test and control lenses, owing to the short visible wavelength filtering of the test lens. Although the differences seen with the two-point light threshold test occurred across age groups, they tended to be more marked in the older age group. For example, when the lens was activated, the average separation for the young subjects (M±SD) was 2.36±1.09 mm (broadband condition) and 5.58±3.2 mm (violet condition). In contrast, the averages for the older group were 2.16±1.22 mm (broadband condition) and 4.95±3.04 mm (violet condition).

### Halo Diameter

**FIG. 3.**
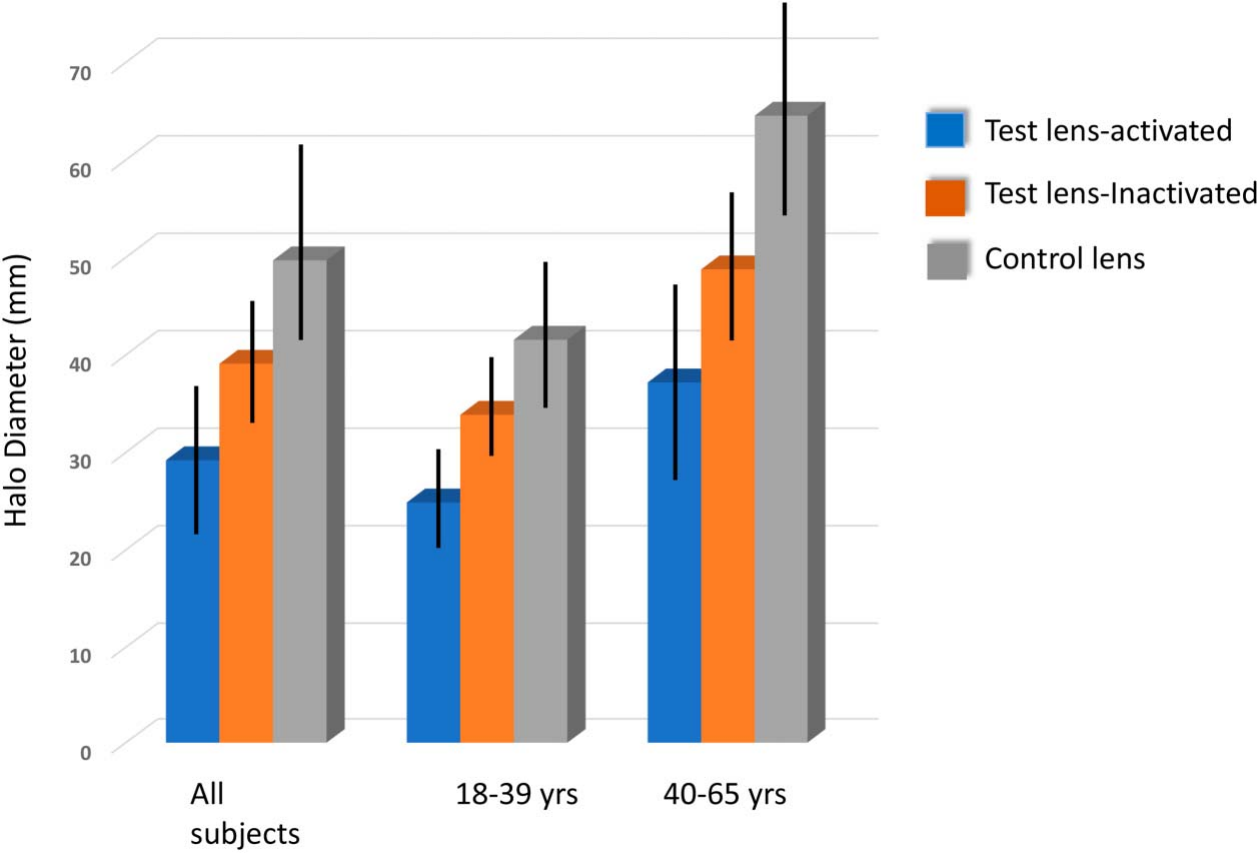
Mean and SD values for the diameter of halos as measured while wearing an activated photochromic contact lens, a control contact lens (under the same activation conditions), and an “inactivated” photochromic (inactivated in this context, means only the activation that would normally accompany changes associated with ocular ambient temperature and regular exposure to the light within the stimulus).

Halo diameter was measured using the control and the test lens with and without an activating light source. In all cases, the photochromic contact lens provided smaller halo diameters than the nonphotochromic lens. In all but one of these scenarios (younger group/inactivated) these differences were statistically significant. Note the overall larger halos observed with the older group, and the 2 to 3 times reduction of halo size while wearing the photochromic lens compared with those observed with the younger group. This is shown in Figure 3.

### Starburst Diameter

The methodology and results for evaluating starbursts were similar to the halo testing. Starburst diameter was measured with and without an activating light source—creating the same four testing scenarios per age group. In all testing scenarios, the photochromic lens resulted in smaller starburst diameters than the nonphotochromic lens, and these differences were all statistically significant. For example, the average diameter of the starbursts for the younger subjects using the activated lens was 39.6±14.8 mm. In contrast, the average for the older subjects was 56.8±33.4 mm. The difference in the magnitude of this effect was statistically significant (*P*=0.001).

## DISCUSSION

The results of this study indicate that wearing a photochromic contact lens (inactivated or activated) leads to reduced signs of dysphotopsia and two-point thresholds for younger and middle-aged adults when compared with a nonphotochromic contact lens assessed in the contra-lateral eye and tested under the same conditions. This difference between the lenses tended to increase with age such that filtering conferred greater improvements. On the face of it, this makes sense: with increasing age comes an increased probability that there may be more ocular issues that need improving (e.g., if the older eye has more scatter, filtering that scatter may result in greater benefit). As a general trend, scatter-related phenomenon do tend to increase with age.^13–15^ Pulling et al. ^16^ measured glare thresholds on 148 subjects ranging from ages 5 to 91 years. He found that, although there was wide variation within a cohort, glare thresholds (measured directly or in a driving simulator) did not decline until about 40 years but after declined rapidly. This raises the question of whether strategic filtering for subjects with higher scatter, say older subjects, would be more useful than for individuals with lower levels of scatter.

There is some evidence that addresses this question. Stringham et al.,^17^ for instance, used lutein and zeaxanthin supplements for 6 months to increase macular pigment filtering in a sample of younger subjects. They showed that glare thresholds did not improve as a simple linear function of MP filtering increases. The largest improvement was at the highest level of filtering and the most intense levels of glare; a 0.16 log increase in MP was related to a 0.22 log change in glare thresholds. An age-stratified effect of filtering on visual performance under glare conditions has also been reported. Mahjoob et al. ^18^ assessed visual acuity and contrast sensitivity under glare conditions in 60 subjects (ages 5–60 years) who wore yellow filtering spectacle lenses. They found that the yellow filters improved vision significantly (*P*=0.007) in only the oldest set of subjects (from 51 to 60 years) (although glare reduced performance at all ages).

Our results are consistent with the hypothesis that filtering by a photochromic contact lens can reduce the signs of dysphotopsia (halos and starbursts) for both younger and middle-aged adults. Subjects with more scatter, particularly older subjects, however, may accrue the most benefit.
